# Taurocholic acid induces intrahepatic cholangiocyte cell proliferation via activating NRAS and YAP1

**DOI:** 10.1371/journal.pone.0339210

**Published:** 2026-02-04

**Authors:** Lum Fube, Arul Thason, Elizabeth Taliaferro, Carol Heckman, Surya Amarachintha

**Affiliations:** 1 Department of Biology, Georgia Southwestern State University, Americus, GeorgiaUnited States of America; 2 Department of Biological Sciences, Bowling Green State University, Bowling Green, Ohio, United States of America; USDA-ARS: USDA Agricultural Research Service, UNITED STATES OF AMERICA

## Abstract

Biliary atresia is a neonatal cholangiopathy characterized by loss of the extrahepatic bile duct causing bile acid accumulation in the liver and subsequent fibro inflammation and abnormal proliferation of intrahepatic ducts. Further, BA liver develops fibrosis, progresses to cirrhosis, and ultimately results in liver failure requiring liver transplantation. NRAS, an oncoprotein, acts as an effector in cholangiocyte cell proliferation and differentiation. We hypothesize that elevated levels of Taurocholic acid (TCA), a conjugated bile acid, activates YAP1 via NRAS, inducing cholangiocyte proliferation. Our experiments using mouse intrahepatic cholangiocyte cells treated with TCA showed significant proliferation of cells at 100 μM compared to control, 10, and 1000 µM. Immunofluorescence staining with KRT19 and EPCAM antibodies showed neither loss of protein expressions nor altered morphology of cholangiocyte cells with TCA treatments suggesting no loss of cholangiocyte integrity. Fluorescent images measured by Image-J showed elevated NRAS and YAP1 expressions in cells treated with 100 µM TCA for two days compared to control. Further, colocalization analysis revealed YAP1 was translocation to the nucleus presumably. There it can act as a transcription factor and induce *TEAD1* expression. In addition, NRAS overexpressed at 100 μM of TCA activated downstream targets *MAPK1*. We conclude that TCA induces abnormal cholangiocyte cell proliferation by triggering NRAS production and causing a downstream activation and translocation of YAP1. However, TCA at lower doses showed no significant impact on cholangiocyte cells but at higher doses caused toxicity and cell death.

## Introduction

Cholangiopathies are a class of complex and progressive liver diseases that target the cholangiocytes, epithelial cells lining the bile ducts in the liver [1]. The most common neonatal cholangiopathy is Biliary Atresia (BA). The disease is characterized by loss of extrahepatic bile duct and inflammation of intrahepatic bile ducts impairing bile drainage, thus leading to the accumulation of bile acids in the liver [2]. This accretion in the liver results in elevated levels of total serum bile acids, inducing acute hepatic inflammation and injury that rapidly progresses into hepatic fibrosis and, eventually, cirrhosis if left untreated [3]. Some common postulations are that the atresia of bile ducts may result because of the developmental defects in bile ducts during pregnancy (suggesting a prenatal genesis of liver dysfunction), viral infection or exposure to environmental toxins at birth activating a deregulated innate and adaptive immune response that amplifies cholangiocyte injury [4–6].

During the immune response, cholangiocytes exhibit cytotoxicity characterized by abnormal cell proliferation and apoptosis – as a function of cell degradation which eventually results in fibrosis, obstruction of the ductal lumen, and bile duct loss [7,8]. Surgical intervention by hepatic portoenterostomy (HPE), known as the Kasai procedure is to restore biliary flow and bile drainage by connecting the bile duct to the Roux-en-Y loop jejunum is the crucial intervention once BA is diagnosed [9,10]. However, the Kasai procedure, in many cases, only aids in slowing the progression of the intrahepatic disease. Post-procedure many patients still deal with the prognosis of cholangitis, a bacterial infection that occurs predominantly in patients after KPE and is commonly treated with antibiotics manifesting in ways such as portal hypertension and advanced cirrhosis [11–13]. Pathogenesis to cirrhosis post-Kasai procedure suggests that subsistent damaging molecular processes are ongoing, although the pathogenic mechanisms by which these occur are ambiguous [11,14]. A prominent causative factor that researchers are studying is the bile acids in those with biliary atresia, particularly the increase in serum levels during the progression of the disease and its correlation with the inflammatory fibrosis stage [11,15]. BA patients who had early diagnosis and HPE showed significantly elevated taurocholate levels in blood (0.98 ± 0.62 µmol/L) compared to normal infants (0.43 ± 0.40 µmol/L) [16].

Bile acid-coenzyme A: amino acid N-acyltransferase (BAAT) is one of the critical liver enzymes responsible for producing conjugated bile acids. Deficiencies in this enzyme lead to abnormal bile acids and are caused by mutations in the gene that encodes it, the peroxisomal protein-encoding gene BAAT found in hepatocytes [17]. Among the possible causes of damage post-Kasai is the oncogene N-RAS, a gene with limited data on its potential involvement in the pathways that lead to the prognosis of the disease. N-RAS is a gene of interest because it has been shown to play a key role in mediating cholangiocyte proinflammatory cytokine production [18]. Evidence is beginning to show that cholangiocytes, initially thought to just be affected by the liver’s proinflammatory immune response to various stresses, are actually an effector [7]. Experimental evidence has shown that cholangiocytes and hepatocytes in BA livers have impaired cell junctions and polarity complexes that are caused by Cdc42 insufficiency resulting in impaired intrahepatic bile ducts [19].

Cholangiocytes’ response to the perpetual exposure of the typical and pathologic substances in bile is normally highly regulated so there is no aberrant response of the immune system. However, the deregulated innate immunity characterized by many cholangiopathies including BA, leads to a response of the cholangiocytes where they can acquire an activated phenotype that expresses an abnormal pattern of immune system proteins such as chemokines, cytokines, and their receptors [7,20]. Toll-like receptor signaling pathway is a key player in the pathological response of the cytokines and chemokines by cholangiocytes and involves the hydrolase enzyme Ras [7]. Ras, a group of small cellular GTPases that regulate diverse cellular processes, has three isoforms – H-Ras, K-Ras, and N-Ras [21]. Recent studies have shown the oncogene N-RAS to be the primary Ras protein expressed in cholangiocytes [7,18]. The brief association of TLRs with lipopolysaccharides in cholangiocytes activates N-Ras and leads to rapid phosphorylation of the downstream Ras effectors extracellular signal-regulated kinases (ERKs) 1 and 2 [7,20].

Although the effect of N-Ras activation in association with liver injury has been widely studied, there is limited knowledge on N-Ras activation in Biliary Atresia. Because of the evidence supporting that BAAT has strong affinity towards taurine over glycine in forming conjugated bile acids [22], loss of extrahepatic bile duct results in the accumulation of bile acid [11], and N-Ras’ role in the proliferation of cholangiocyte cells [23], we hypothesize that the accumulation of conjugated bile acids triggers the N-Ras proliferation pathway, and inhibition of this pathway could help achieve a better prognosis of BA post-Kasai procedure. In this study, we aim to build on the work of previous studies and evaluate the effect of N-Ras inhibition by various chemical treatments in improving cholangiocyte function.

## Materials and methods

### Processing of RNAseq datasets

Public and freely available RNAseq data (GSE186444) of cholangiocyte organoids (COs) generated from livers of normal (n = 3) and biliary atresia (BA) patients at diagnosis (Dx, n = 5) and transplant (Tx, n = 6) were obtained from GEO database [4]. Comparisons between BACO-Dx and BACO-Tx were performed using GEO2R (https://www.ncbi.nlm.nih.gov/geo/geo2r/) to identify differentially regulated gene expression profiles. ToppGene Suite was applied to the genes UP-regulated in BACO-Tx compared to BACO-Dx to prioritize biological process based on their functional similarity [24].

### Mouse cholangiocyte cell line cultures

Cholangiocyte cell line derived from mouse intrahepatic bile ducts were generously provided by Dr. Heather Francis, Indiana University, USA [25,26]. Briefly, cells were cultured as adherent cells using Dulbecco’s Modification of Eagle’s Medium (DMEM) (Corning, cat# MT10014CV) supplemented with 10% heat-inactivated Fetal Bovine Serum (Corning, cat# MT35017CV) and 1% Penicillin-Streptomycin (10,000 U/mL) (Gibco, cat# 15140122). Cells were cultured in a humidified incubator supplied with 5% CO_2_ and maintained at 37°C. To expand the cultures, adherent cells were treated with 0.05% Trysin-EDTA (Gibco, cat# 25300054) for 8 minutes before they were harvested.

### Taurocholic acid treatments

Cultured cholangiocyte cells were treated with TCA (MilliporeSigma, cat# 580218) at different concentrations and tested for different time intervals. TCA concentrations at 10 μM, 100 μM, and 1000 μM were tested over 24 hrs or 48 hrs. Untreated cells served as control while experiments were conducted a minimum of three independent times.

### Immunofluorescence of cell cultures

Cholangiocyte cells were plated in glass bottom dishes (ThermoScientific, cat# 12567400) before the experiments were performed. Cells either untreated or treated with TCA were washed twice with 1X PBS and fixed for 10 minutes using 4% paraformaldehyde (Electron Microscopy Sciences, cat# 15710) made in 1X PBS at room temp. Blocking buffer constituted of 1% bovine serum albumin (Fisher BioReagents, cat# BP1600), 5% donkey serum (Jackson Immuno Research Labs, cat# NC9624464) and 0.1% Triton X-100 Surfactant (MilliporeSigma, cat# MTX15683) made in 1X PBS was added to the cells at room temperature (RT) for one hour. Primary antibodies (S1 Table) were diluted to the appropriate concentrations, based on manufacturer’s recommendations. in blocking buffer and cells were incubated overnight at 4°C. Matching secondary antibodies (S2 Table) were diluted, and cells were incubated for one hr at RT. To stain the nuclei, cells were incubated with Hoechst (Invitrogen, cat# H1398) diluted to 1 in 8000 1X PBS for ten minutes. Slides were mounted with Fluoromount (Invitrogen, cat# 5018788) and allowed to sit overnight at RT in dark. Images were captured using Nikon fluorescence microscope fitted with 18mega pixels color CMOS C-Mount microscope camera equipped with reduction lens (Amscope, cat# MU1803). Captured images were processed using ImageJ software to obtain mean gray values of fluorescent intensity and saved at 600 pixelsperinch high resolution tiff files.

### Gene expression by qPCR

#### RNA Isolation.

An RNAqueous kit (Invitrogen, cat# AM1912) was used to isolate total RNA from the cells grown in 100 mm culture dishes, plated at a density of 2 x 10^6^ cells per dish. Cells either untreated or treated with TCA were washed with 1X PBS to remove traces of medium and were lysed using lysis binding solution provided with the kit. Lysate was recovered in test tubes and equal volumes of 65% ethanol were added with gentle mixing by vortexing. The lysate ethanol mixture was then loaded to a collection tube and centrifuged at RCF 10,000X for 45 seconds. The flow-through was discarded and the filtrate was washed with different wash solutions that were supplied in the kit.

The filter cartridge was then put into a fresh collection tube, and preheated elution solution was pipetted to the center of the filter and the tube centrifugated for 30 seconds, after which the eluate was recovered. This step was repeated to ensure all the RNA was recovered from the samples.

#### cDNA synthesis and PCR.

complementary DNA synthesis was performed using RevertAid First Strand cDNA Synthesis Kit (ThermoScientific, cat# FERK1622). cDNA synthesis, PCR amplification of first stand cDNA, control PCR amplification, and gel electrophoresis were performed as follows.

**cDNA synthesis:** Following reagents were added to a sterile, nuclease-free tube kept on ice in the following order: Total RNA (0.1 ng – 5 μg), Oligo (dT)18 primer (1 μl), and 12 μl of nuclease free water. To this solution, 4 μl of 5X reaction buffer, 1 μl of RiboLock RNase inhibitor (20 U/μl), 2 μl of 10 mM dNTP Mix, and 1 μl of RevertAid M-MuLV RT (200 U/μl) was added. This solution was then incubated for 60 minutes at 42 ºC in a thermal cycler (BioRad T100). The reaction was terminated by heating to 70 ºC for 5 minutes.

**Gene amplification by qPCR:** cDNA was diluted to 1:1000 in nuclease free water. A thin-walled PCR strip tube was put on ice and the following reagents added: 2 μl of diluted cDNA, 5 μl of 10X PCR buffer, 1 μl (0.2 mM each) of 10 Mm dNTP mix, 3 μl of 25 mM MgCl2, 0.5 μl each of Forward and Reverse Primers, 0.5 μl of Taq DNA polymerase (5 U/ μl), and 35.5 μl of nuclease free water. Primer sequences for mouse genes specified in the S3 Table were retrieved from the PrimerBank database, a public resource for PCR primers that are experimentally validated and can be used to detect and quantify gene expressions [27].

**Agarose gel electrophoresis:** 1% agarose gel was prepared with 1xTris-Acetate-EDTA (TAE) buffer (Fisher BioReagents, cat# BP13354). The agarose solution was mixed with 5 μl of SYBR (Invitrogen, cat# S33102) safe to allow the binding of DNA and visualization under ultraviolet light. The gels were loaded with PCR mix and run for 30 minutes at 120 V using a compact power supply (Owl™ EC300XL2).

### Statistical analysis

Cell numbers from cell proliferation assays and mean gray values for fluorescent images from Image J were analyzed using t test or ANOVA using GraphPad Prism software. Significance between untreated and treated samples was determined using an unpaired t test or one-way ANOVA. Data in figures were expressed as mean ± SD (standard deviation) and p values of <0.05 were considered statistically significant. All data presented were generated from three or more independent experiments.

## Results

### Cholangiocyte organoids revealed elevated expressions of NRAS and YAP1

**Fig 1:**
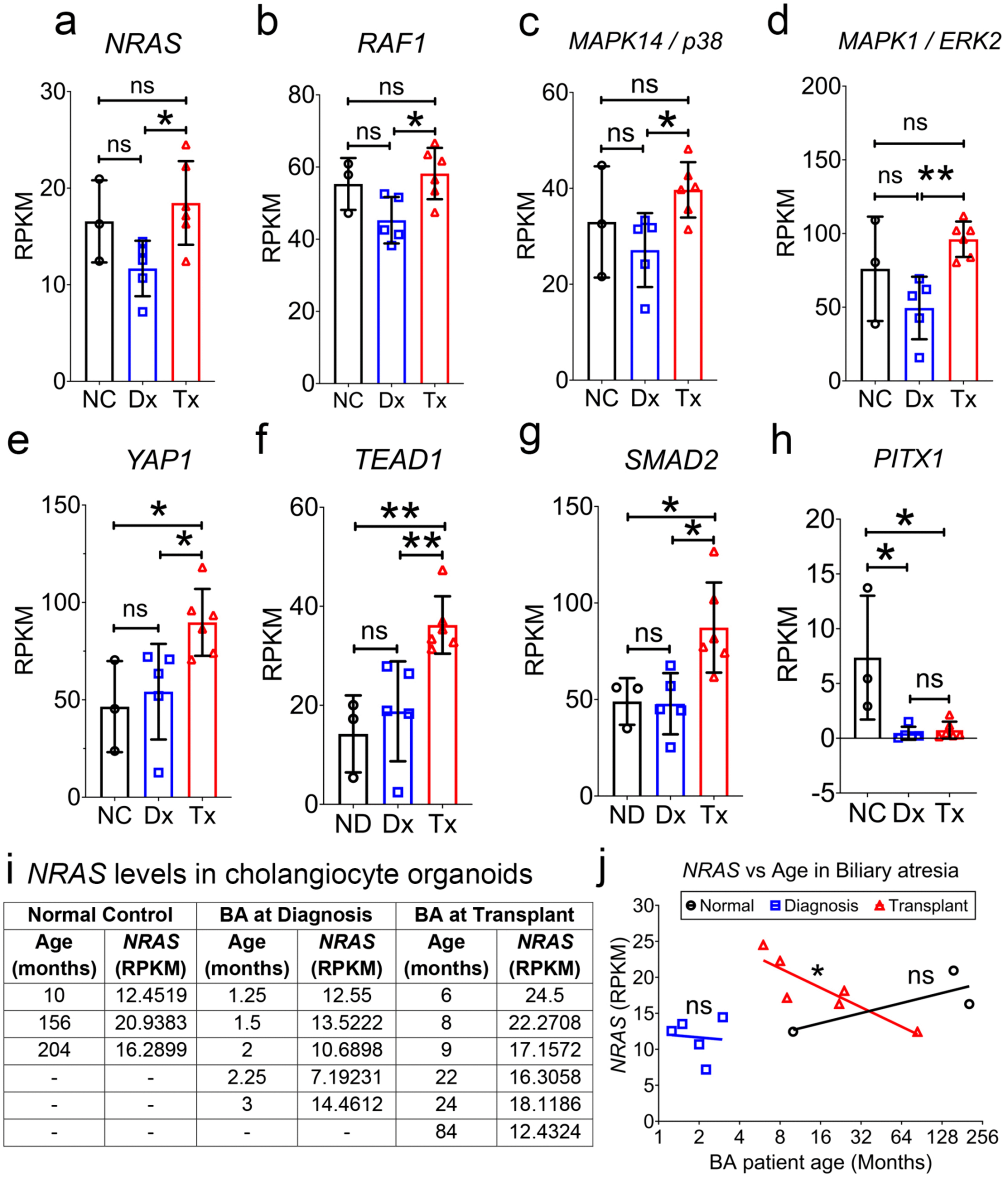
Gene expressions from previously published bulk RNA-seq of cholangiocyte organoids generated from human livers of normal controls (NC) and biliary atresia (BA) at diagnosis (Dx) or transplant (Tx). **a.** NRAS, neuroblastoma ras oncogene; **b.** RAF1, Raf-1 proto-oncogene, serine/threonine kinase; **c.** MAPK14/p38, mitogen activated protein kinase 14; **d.** MAPK1/ERK2, mitogen-activated protein kinase 1. **e.** YAP1, Yes1 associated transcriptional regulator; **f.** TEAD1, TEA domain transcription factor 1; **g.** SMAD2, SMAD family member 2; **h.** PITX1, paired like homeodomain 1. **i.** Tabular display of *NRAS* gene expression levels in cholangiocyte organoids generated from livers of Normal donors, BA patients at diagnosis and transplant. **j.** Simple linear regression of NRAS expression levels plotted against age for normal, BA patients at diagnosis and transplant, data was analyzed by Pearson correlation. RPKM, Reads Per Kilobase Million Mapped Reads. Data is represented as mean ± SD. ns. = non-significant, * p < 0.05, ** p < 0.001.

To identify gene signatures that were significantly altered in response to the elevated conjugated bile acids in BA, we analyzed publicly available curated gene expression datasets (GSE186444) of cholangiocyte organoids (CO) which were generated from livers of normal control (NC) and BA patients at diagnosis (Dx) or transplant (Tx) (S1, S2 File). Using ToppGene, functional enrichment of gene sets that were up-regulated in BACO-Tx compared to BACO-Dx identified “cell cycle” as a top biological process (S3 File). Next, comparing genes across COs we identified expressions of *NRAS* and *YAP1* significantly upregulated (Fig 1a,1e) but for *PITX1* downregulated (Fig 1h). Subsequently, using pathway enrichment analysis for these three genes, we identified NRAS downstream effectors, RAF1, *MAPK14/p38*, and *ERK2* (Fig 1b–1d) and *YAP1* downstream targets *TEAD1*, and *SMAD2* (Fig 1f,1g) significantly upregulated but not for *PITX1*. To determine if there is a relationship between activation levels of NRAS and age of BA patients at diagnosis and transplant, we compared the gene expression levels across five diagnosis and six transplant patients. Our analysis revealed no significant correlation among patients at diagnosis; however, transplant patients showed a significant inverse correlation with age (Fig 1i,1j), indicating patients who came for early transplant had higher NRAS levels. This suggested activation of NRAS pathway in Tx compared to Dx (see Discussion).

### Exposure to taurocholic acid induced cholangiocyte cell proliferation

**Fig 2:**
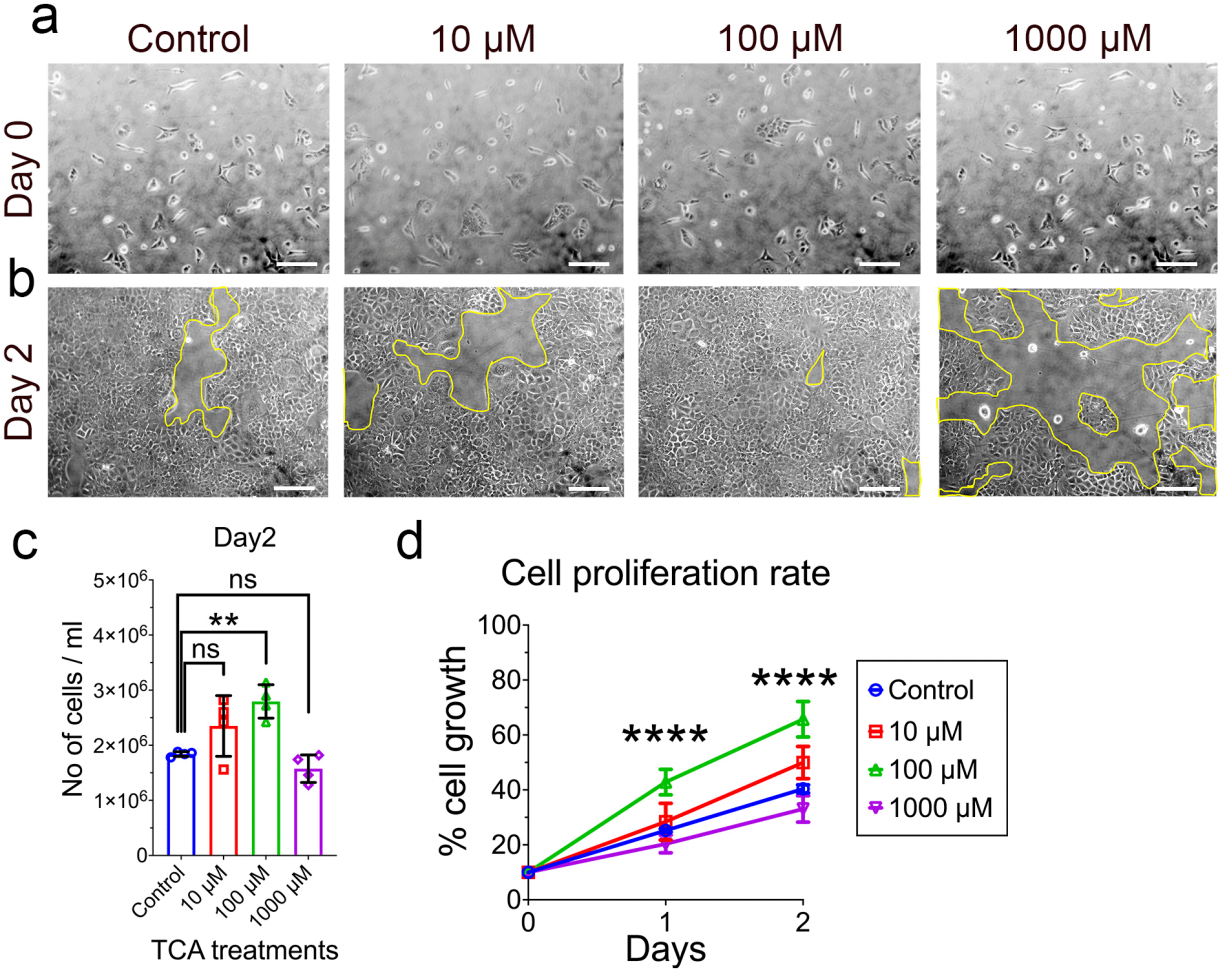
a. Phase contrast images of mouse cholangiocyte cells on day 0. **b.** cells were either untreated (control) or treated for two days with 10, 100, and 1000μM concentrations of Taurocholic acid (TCA). Yellow lines circle the space devoid of cells in the dish. The scale bar represents 50μm. **c.** Quantification of total number of viable cells counted using trypan blue exclusion assay after cells were untreated or treated with TCA for two days. **d.** Cell proliferation rates of viable cells either untreated or treated with TCA for days 1 and 2 were calculated based on day 0 and plotted as percentages. Data is represented as mean ± SD., N = 4, ns. = non-significant, ** p < 0.01, **** p < 0.0001.

To test the hypothesis that “accumulated bile acids in BA livers induce cholangiocyte proliferation via NRAS and YAP1 pathways”, an experimental model of the disease was established by treating the mouse cholangiocyte cells with TCA. TCA is a conjugated bile acid known for its toxicity in the liver and is one of the bile acids with elevated levels in cirrhosis. To determine the concentration that would abnormally increase the cell proliferation, the experimental group was treated with a low dose of TCA (10 μM), a medium dose (100 μM), and a high dose (1000 μM). Mouse intrahepatic cholangiocyte cells plated at equal densities (Fig 2a) were exposed to TCA for two days (Fig 2b). We observed that at the end of two-day treatment, cells exposed to 100 μM TCA had a significantly higher cell count compared to the control, however, 10 μM and 1000 μM showed no significant difference in growth (Fig 2c). When proliferation rates for 24 and 48 hrs. were plotted, a significant steady growth with 100 µM treatments was observed but not with other doses (Fig 2d). Furthermore, another set of experiments were performed to determine the cytotoxicity of TCA. Cells were grown to confluent and subjected to TCA treatments at 10 µM and 100 µM, and 1000 µM for 24 hrs. As shown in the supplemental figure (S1 Fig), cells with 100 µM continued to grow at significant rates compared to control (untreated), however, 10 µM showed no significant (difference in) growth compared to control whereas 1000 µM showed partial cell death indicating toxicity. We conclude that low dose of TCA is not sufficient to activate the NRAS and YAP1 pathways while high doses may have triggered inhibitory pathways because of toxicity.

### Taurocholic acid treated cells retained cholangiocyte integrity

Cytokeratin 19 (KRT19) and Epithelial cell adhesion molecule (EPCAM) are characteristic markers of mature cholangiocyte cells and epithelial cells and are expressed in cytoplasm and cell membrane of biliary epithelial cells respectively. Before proceeding with further experiments, cells were characterized by detecting cholangiocyte markers after TCA treatments. This is to demonstrate whether cells maintained their epithelial integrity without undergoing epithelial to mesenchymal transition (EMT) as indicated by the loss of KRT19 and EPCAM. The cells were treated with 100 µM since there was significant change in cell proliferation and 1000 µM which induced no change in cell number. Immunofluorescence imaging showed CK19 expressions levels increased significantly with 1000 µM (S2 Fig). Although EPCAM levels dropped significantly with 1000 µM, the protein expression was still visible (S3 Fig). This demonstrated that TCA did not transcriptionally reprogram cholangiocyte cells resulting in EMT when treated with 100 or 1000 µM.

### Cell proliferation markers were induced with TCA treatment

**Fig 3:**
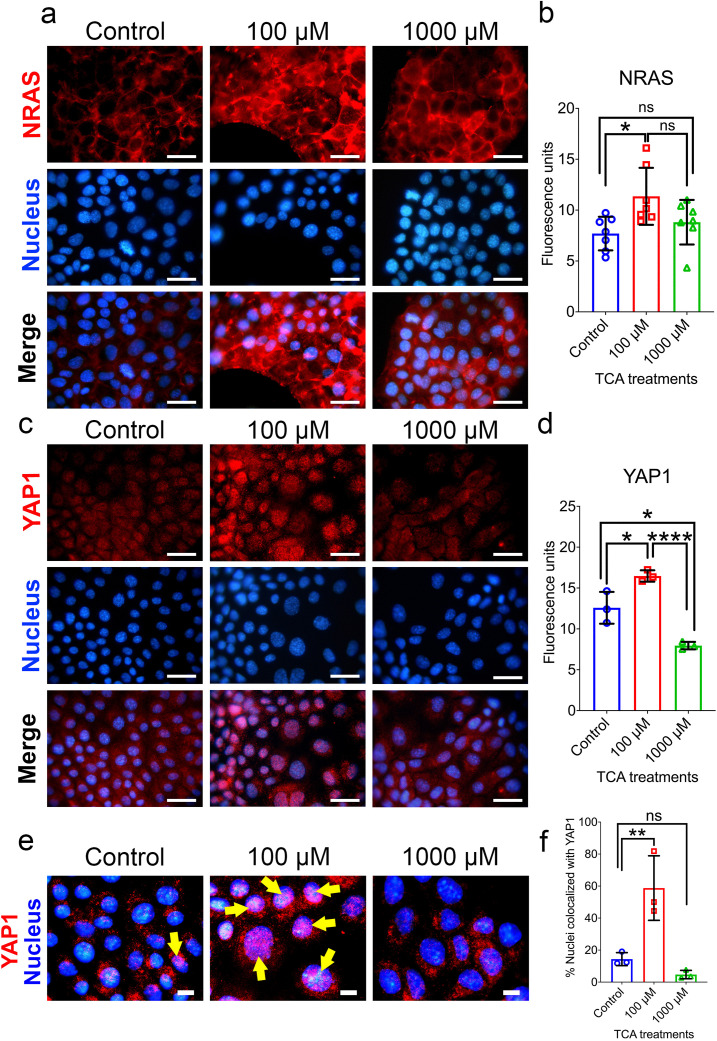
Immunofluorescent staining for NRAS (a) and YAP1 (c) in cholangiocyte cells either untreated (Control) or treated with 100μM and 1000 μM taurocholic acid (TCA) for two days. The scale bar represents 20μm. Quantification of fluorescence intensity in red for NRAS (b) and YAP1 (d) was measured by image J and averaged by number of nuclei (blue). (e) Fluorescence Images were analyzed for colocalization of YAP1 (red) with nucleus (blue) as depicted by yellow arrows. The scale bar represents 10μm. (f) Quantification of nuclei colocalized with YAP1. Data is represented as mean ± SD., N = 3, ns. = non-significant, * p ≤ 0.05, ** p ≤ 0.01, **** p ≤ 0.0001.

**Fig 4:**
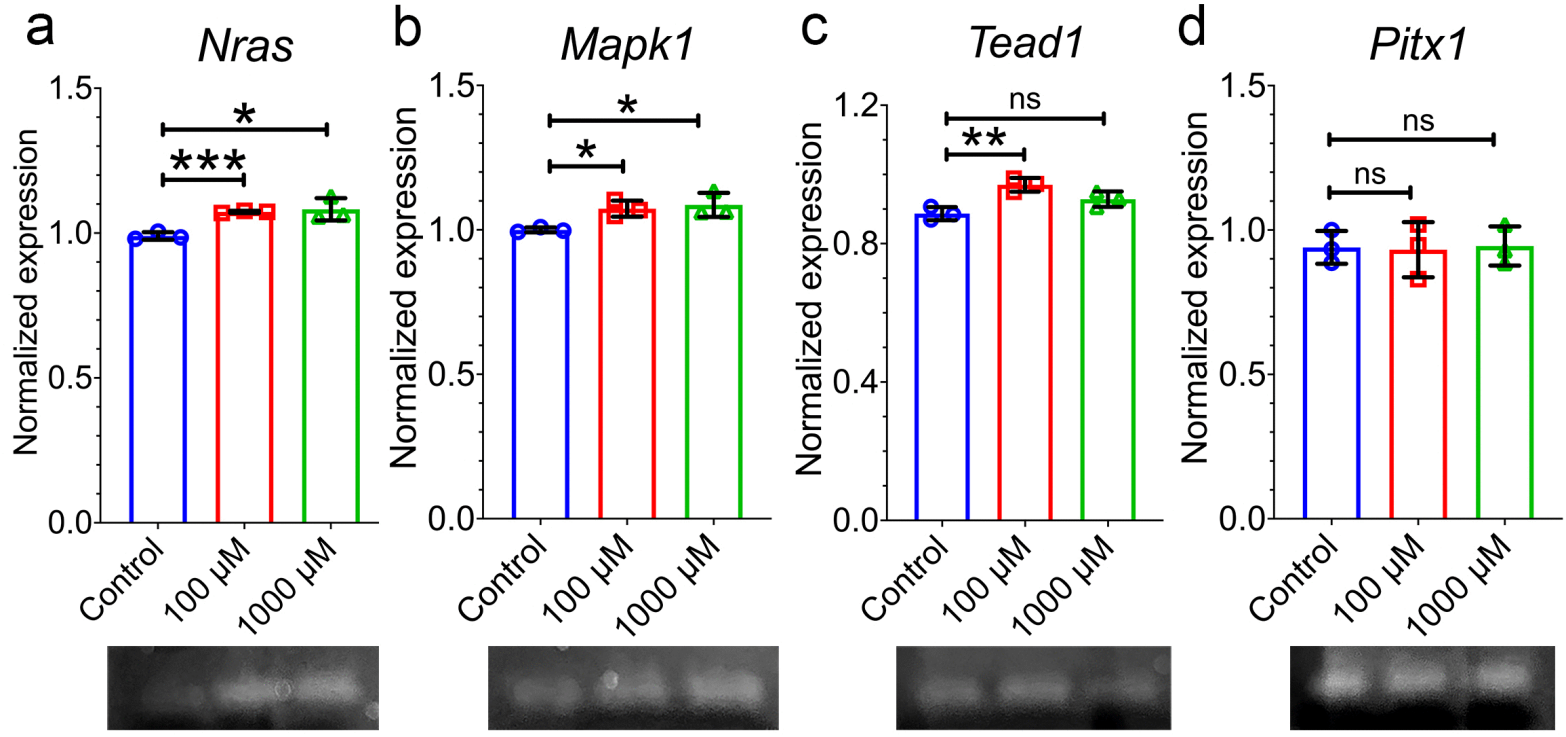
qPCR for genes involved downstream in cell proliferative pathways of cholangiocyte cells either untreated or treated with TCA at 100 uM and 1000 uM for two days. Image J software was used to analyze the intensity of bands. Genes *Nras* (neuroblastoma ras oncogene), *Mapk1* (mitogen-activated protein kinase 1), *Tead1*(TEA domain transcription factor 1), *Pitx1* (paired-like homeodomain transcription factor 1) were normalized to *Gapdh*. Below each paneal are images of PCR products that were subjected to agarose gel electrophoresis bands. Data is represented as mean ± SD. ns. = non-significant, * p < 0.05, ** p < 0.01, *** p < 0.001.

**Fig 5:**
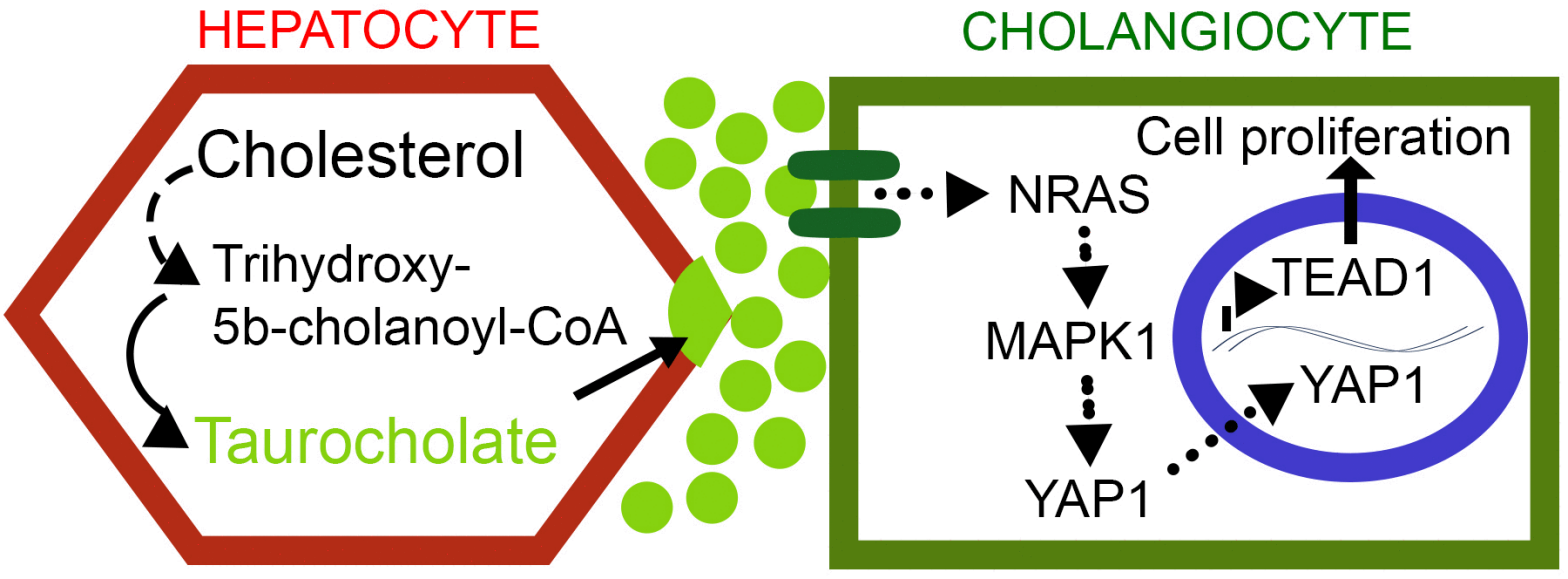
Schematic representation of cholangiocyte cell proliferation in Biliary atresia via NRAS and YAP1 activation triggered by accumulation of Taurocholic acid that ultimately results in intrahepatic ductular reaction. BAAT, bile acid-CoA:amino acid N-acyltransferase; TEAD1, TEA domain transcription factor 1; YAP1, Yes1 associated transcriptional regulator; NRAS, neuroblastoma ras oncogene; MAPK1, mitogen-activated protein kinase 1. (• • ►, indirect activation; → direct activation).

N-RAS, localized to cytosol, is known for its role in cell cycle division and when activated becomes an effector in an abnormal cell proliferation pathway. When cells were treated with 100 μM and 1000 μM for two days and subjected to immunofluorescence with antibody for NRAS, they demonstrated significantly elevated levels in 100 μM but non-significant in 1000 μM compared to untreated (Fig 3a and 3b). YAP1 is localized to cytosol when inactive, however, when turned ON, YAP1 moves to the nucleus where it acts as a transcription factor. Upon translocation, YAP1 controls genes that promote cell proliferation. Immunofluorescence staining for YAP1 in control, 100 μM, and 1000 μM treatments detected low levels of protein expression in control and 1000 μM treated cells with cytosolic localization. However, in the 100 μM-treated cultures, there was a much higher expression of YAP1 (Fig 3c and 3d), and it was partially localized in the nucleus (Fig 3e). To further investigate whether the activation of NRAS and YAP1 induced downstream target, *Mapk1* and *Tead1* respectively were examined for gene expression levels using quantitative PCR technique. Results showed that 100 μM treatment significantly induced the genes, however, only *Mapk1* but not *Tead1* were induced with 1000 μM. Further, *Pitx1* expression was also determined to see if it was acting upstream of NRAS. However, no significant changes were observed indicating *Pitx1* may not be involved in the cell proliferation pathway (Fig 4a, 4b, 4c, and 4d). In summary, we were able to establish a cell proliferation model with 100 μM TCA treatment and to demonstrate that NRAS was activated. This can indirectly trigger YAP1, which translocates to nucleus and upregulates the expression of *TEAD1* resulting in cell proliferation (Fig 5).

## Discussion

Comparing the gene expressions of COs generated from human livers of NC, BA at diagnosis, and BA at transplant, we found significant differences between diagnosis and transplanted groups in *YAP1*, *NRAS*, and their downstream effectors. Further, significant differences between normal and transplanted groups are observed only in *YAP1* and its downstream effectors. In contrast, there were no significant differences between control and diagnosis groups for any of the genes analyzed. We believe that COs generated from the livers of diagnosis patients were not significantly damaged. This is also supported by the evidence that Kasai procedure performed in BA patients before 45 days of age and received high-dose steroids showed improvement in native liver survival [28].

In this study, we aimed to build on the work of previous studies to evaluate the effect of Hippo/YAP pathway activation via N-Ras, induced by taurocholic acid treatment [29]. While low concentrations of TCA had no significant effect on cholangiocytes, moderate concentrations had profound impact on cell proliferation. Similarly, our observation is in accordance with a recent study that measured serum bile acids to predict liver injury post-KPE and demonstrated that serum cholic acid fractions positively correlated with ductular reaction [30]. Conversely, high concentrations of TCA proved toxic to the cholangiocyte cells and is comparable to another study that developed serum bile acids as a prognostic biomarker in BA. In their study, 6‐month post‐KPE patients with >40 μmol/L serum total bile acids, 42.9% of them had 10-year cumulative incidence of liver transplant/death while 64% developed clinically evident portal hypertension [31]. In BA patients, although there is ductular reaction (DR) often there are no patent bile ducts visible in transplant livers. DR predicted poor native liver survival and rapid liver fibrosis. In association with serum total bile acids DR had a strong correlation in predicting different stages of the disease before transplantation [32]. We sought to test the hypothesis that TCA would induce the expressions of NRAS and YAP1, proteins when activated are associated with cholangiocyte cell proliferation [18,33].

Our results demonstrated that cells proliferated rapidly with 100 µM and developed abnormal protein levels of NRAS and YAP1. Further, there were significant levels of YAP1 present in the nucleus. MAPK1 is known to be coactivated with NRAS, while TEAD1 is transcriptionally activated by YAP1, a transcriptional factor that controls genes involved in cell proliferation [33–35]. *PITX1* is involved in several cancers however, its role in cholangiocarcinoma is still unclear. A previous study has demonstrated that PITX1 can serve as early diagnosis marker for hepatocellular carcinoma, however, PITX1 downregulates RAS activity suppressing tumor formation [36,37]. In this study, we were able to validate abnormally high gene expressions of *MAPK1* and *TEAD1*, however, we did not see any significant change in *PITX1*. Taken together, our results indicate that treatment of cholangiocytes with 100 μM TCA demonstrated cell proliferation and induced YAP1 expression which can be related to ductular reaction and progression of liver damage in BA and may serve as *in vitro* model for BA [21,29]. However, *in vivo* studies using experimental BA mouse models or human cholangiocytes are required to further validate translational relevance. To capture further insights into whether NRAS and YAP1 signatures were unique to BA transplanted group, we examined primary sclerosing cholangitis (PSC) transplanted group, a chronic liver disease with similar degree of fibrosis. Extrahepatic cholangiocyte organoids (ECOs) generated from PSC patients revealed genes for mucosal maintenance, hypoxia, reactive oxygen species, and long noncoding RNA upregulated but not cell proliferation with NRAS and YAP1 [38]. Taken together, the data suggests that BA transplanted group although is heterogenous, compared to diagnosis group NRAS and YAP1 pathways are upregulated and unique to BA.

All in all, our bioinformatic analysis using RNAseq data from cholangiocyte organoids and *in vitro* studies using cholangiocytes identified NRAS/ YAP1 pathway triggered by TCA, may serve as novel early diagnosis markers of progression to liver failure in biliary atresia.

## Supporting information

S1 Fig Phase contrast images of mouse cholangiocyte cells either untreated (control) or treated with 10, 100, and 1000µM concentrations of Taurocholic acid (TCA). (PDF)

S2. Fig Immunofluorescent staining for Cytokeratin 19 (CK19) in cholangiocyte cells either untreated or treated with 100 µM and 1000 µM TCA for two days. (PDF)

S3 Fig Immunofluorescence staining for Epithelial cell adhesion molecule (EpCAM) in cholangiocyte cells either untreated or treated with 100 uM and 1000 uM TCA for two days. (PDF)

S1 Table List of Primary Antibodies. (DOCX)

S2 Table List of Secondary Antibodies. (DOCX)

S3 Table List of qPCR primer sequences. (DOCX)

S1 File UP regulated genes in BACO Transplant. Genes UP-regulated in Biliary atresia Cholangiocyte Organoids generated from Transplant Liver (BACO-Tx) compared to BACO generated from livers at Diagnosis (BACO-Dx) – GSE186444.
(XLSX)

S2 File DOWN regulated genes in BACO Transplant. Genes DOWN-regulated in Biliary atresia Cholangiocyte Organoids generated from Transplant Liver (BACO-Tx) compared to BACO generated from livers at Diagnosis (BACO-Dx) – GSE186444.
(XLSX)

S3 File TOP Biological Processes in BACO Transplant. From UP regulated genes in BACO Transplant.
(XLSX)
