# Supplementary material for: Taurocholic acid induces intrahepatic cholangiocyte cell proliferation via activating NRAS and YAP1
Source: PLoS One. 2026 Feb 4;21(2):e0339210. doi: 10.1371/journal.pone.0339210 (PMC12871985; doi:10.1371/journal.pone.0339210)
Supplement: S1 Fig — (PDF) [file pone.0339210.s001.pdf]

S1 Fig.

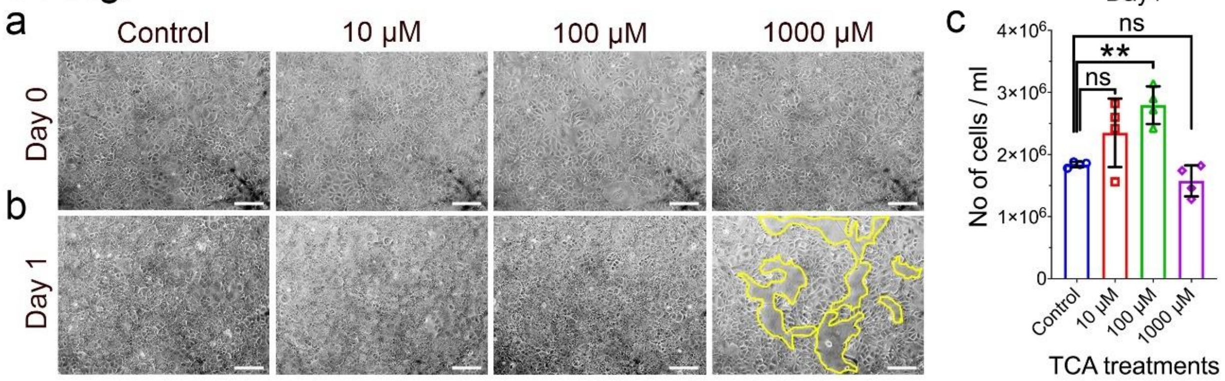

S1 Fig. Phase contrast images of mouse cholangiocyte cells either untreated (control) or treated with 10, 100, and 1000μM concentrations of Taurocholic acid (TCA).
a. Cells grown to complete confluence at Day 0 were followed by untreated (control) or treated with 10, 100, and 1000μM concentrations of Taurocholic acid (TCA). **b.** Cell proliferation after Day 1 of treatment with 10μM and 100μM, however cell death with 1000μM TCA treatments. Yellow lines circle the space devoid of cells in the dish. The scale bar represents 50μm. **c.** Quantification of total number of viable cells counted using trypan blue exclusion assay after cells were untreated or treated with TCA for one day. Data is represented as mean ± SD., N = 4, ns. = non-significant, **** p≤0.0001.
