# Supplementary material for: Taurocholic acid induces intrahepatic cholangiocyte cell proliferation via activating NRAS and YAP1
Source: PLoS One. 2026 Feb 4;21(2):e0339210. doi: 10.1371/journal.pone.0339210 (PMC12871985; doi:10.1371/journal.pone.0339210)
Supplement: S2. Fig — (PDF) [file pone.0339210.s002.pdf]

S2 Fig.

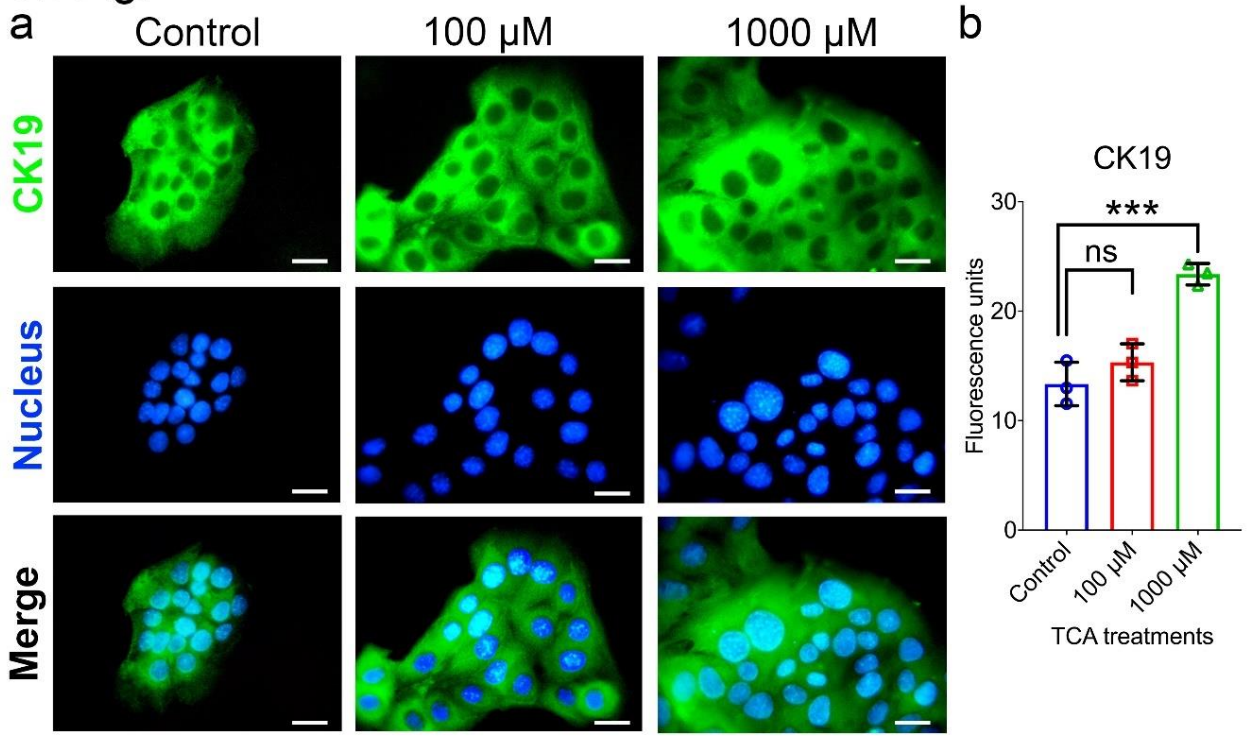

S2. Fig. Immunofluorescent staining for Cytokeratin 19 (CK19) in cholangiocyte cells either untreated or treated with 100 µM and 1000 µM TCA for two days. The scale bar represents 20µm. Graph on right shows elevated levels of CK19 observed with 1000 µM TCA treatment compared to untreated and 100 µm. Data is represented as mean ± SD. ns. non-significant, *** p≤0.001.
