# Supplementary material for: Taurocholic acid induces intrahepatic cholangiocyte cell proliferation via activating NRAS and YAP1
Source: PLoS One. 2026 Feb 4;21(2):e0339210. doi: 10.1371/journal.pone.0339210 (PMC12871985; doi:10.1371/journal.pone.0339210)
Supplement: S3 Fig — (PDF) [file pone.0339210.s003.pdf]

S3 Fig.

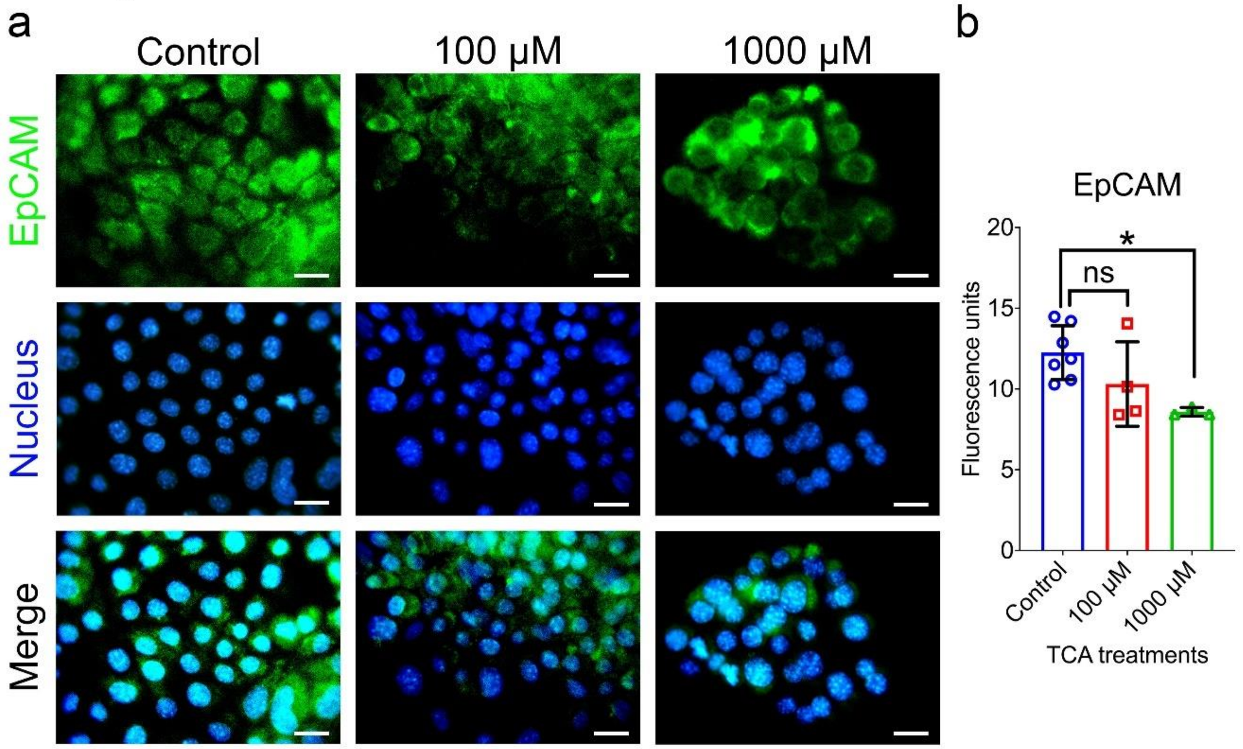


**S3 Fig. Immunofluorescence staining for Epithelial cell adhesion molecule (EpCAM) in cholangiocyte cells either untreated or treated with 100 uM and 1000 uM TCA for two days.** The scale bar represents 20µm. Graph on right for EpCAM shows downregulation with 1000 µM. Data is represented as mean ± SD. ns. non-significant, * $p \leq 0.05$.
