# Supplementary material for: Taurocholic acid induces intrahepatic cholangiocyte cell proliferation via activating NRAS and YAP1
Source: PLoS One. 2026 Feb 4;21(2):e0339210. doi: 10.1371/journal.pone.0339210 (PMC12871985; doi:10.1371/journal.pone.0339210)
Supplement: S1 Table — (DOCX) [file pone.0339210.s004.docx]

**S1 Table. List of Primary Antibodies**

|  | **Antibody** | **Dilution** | **Manufacturer** | **Catalog #** |
| --- | --- | --- | --- | --- |
| **1** | YAP1 | 1:50 | DSHB | YAP1 8J19 |
| **2** | NRAS | 1:500 | Abcam | ab198820 |
| **3** | EPCAM | 1:500 | DSHB | G8.8 |
| **4** | KRT19 | 1:50 | DSHB | TROMA III |

YAP1, Yes1 associated transcriptional regulator; NRAS, NRAS proto-oncogene; EPCAM, epithelial cell adhesion molecule; KRT19, keratin 19.
