# Supplementary material for: Taurocholic acid induces intrahepatic cholangiocyte cell proliferation via activating NRAS and YAP1
Source: PLoS One. 2026 Feb 4;21(2):e0339210. doi: 10.1371/journal.pone.0339210 (PMC12871985; doi:10.1371/journal.pone.0339210)
Supplement: S2 Table — (DOCX) [file pone.0339210.s005.docx]

**S2 Table. List of Secondary Antibodies**

|  | **Antibody** | **Dilution** | **Manufacturer** | **Catalog #** |
| --- | --- | --- | --- | --- |
| **1** | 488 Donkey anti Mouse | 1:1000 | Jackson Immunoresearch | 715-545-150 |
| **2** | 594 Donkey anti Mouse | 1:1000 | Jackson Immunoresearch | 715-585-150 |
| **3** | 594 Donkey anti Rat | 1:1000 | Jackson Immunoresearch | 712-585-150 |
