# Supplementary material for: Taurocholic acid induces intrahepatic cholangiocyte cell proliferation via activating NRAS and YAP1
Source: PLoS One. 2026 Feb 4;21(2):e0339210. doi: 10.1371/journal.pone.0339210 (PMC12871985; doi:10.1371/journal.pone.0339210)
Supplement: S3 Table — (DOCX) [file pone.0339210.s006.docx]

**S3 Table. List of qPCR primer sequences**

| **Mouse gene** | **5’ to 3’** | **Sequence (5’ → 3’)** | **Length** | **Tm (°C)** | **Location** |
| --- | --- | --- | --- | --- | --- |
| ***Nras*** | Forward | ACTGAGTACAAACTGGTGGTGG | 22 | 61.8 | 4-25 |
|  | Reverse | TCGGTAAGAATCCTCTATGGTGG | 23 | 60.7 | 123-101 |
| ***Mapk1*** | Forward | GGTTGTTCCCAAATGCTGACT | 21 | 60.8 | 824-844 |
|  | Reverse | CAACTTCAATCCTCTTGTGAGGG | 23 | 60.8 | 907-885 |
| ***Tead1*** | Forward | AAGCTGAAGGTAACAAGCATGG | 22 | 60.8 | 277-298 |
|  | Reverse | GCTGACGTAGGCTCAAACCC | 20 | 62.8 | 560-541 |
| ***Pitx1*** | Forward | GCCTTCAAGGGAGGCATGAG | 20 | 62.6 | 7-26 |
|  | Reverse | GATTCGCTGGCGGAGTTCTC | 20 | 62.9 | 149-130 |
| ***Gapdh*** | Forward | AGGTCGGTGTGAACGGATTTG | 21 | 62.6 | 8-28 |
|  | Reverse | TGTAGACCATGTAGTTGAGGTCA | 23 | 60.2 | 130-108 |

qPCR Primer sequences for mouse genes specific to induction of cell proliferation along with primer lengths, melting temperatures (Tm), and locations (forward and reverse) on nucleotide sequence. ***Nras***, neuroblastoma ras oncogene; ***Mapk1***, mitogen-activated protein kinase 1; ***Tead1***, TEA domain transcription factor 1**, *Pitx1***, paired-like homeodomain transcription factor 1; ***Gapdh***, glyceraldehyde-3-phosphate dehydrogenase. Primers were diluted in nuclease-free water and used at 200 nM per reaction.
